# Emergency Department Length of Stay Is Associated with Delirium in Older Adults

**DOI:** 10.5811/westjem.59383

**Published:** 2023-05-03

**Authors:** Natalie M Elder, Bryn E Mumma, Meg Y Maeda, Daniel J. Tancredi, Katren R Tyler

**Affiliations:** * University of California Davis School of Medicine, Department of Emergency Medicine, Sacramento, California; † University of California Davis School of Medicine, Department of Pediatrics, Sacramento, California; ‡ The Robert Larner College of Medicine at The University of Vermont, Department of Emergency Medicine, Burlington, Vermont

## Abstract

**Introduction:**

Incident delirium in older patients is associated with prolonged hospitalization and mortality. A recent study suggested an association between emergency department (ED) length of stay (LOS), time in ED hallways, and incident delirium. In this study we further evaluated the emerging association between incident delirium with ED LOS, time in ED hallways, and number of non-clinical patient moves in the ED.

**Methods:**

We performed this retrospective cohort study at a single, urban, academic medical center. All data were extracted from the electronic health record. We included patients aged ≥65 years presenting to the ED and admitted to family or internal medicine services over a two-year period. Patients admitted to any other service, transferred from another hospital, discharged from the ED, or who underwent procedural sedation were excluded. The primary outcome was incident delirium, defined as a positive delirium screen, receipt of sedative medications, or use of physical restraints. Multivariable logistic regression models including age, gender, language, history of dementia, Elixhauser Comorbidity Index, number of non-clinical patient moves within the ED, total time spent in the ED hallway, and ED LOS were fitted.

**Results:**

We studied 5,886 patients ≥65 years of age; median age was 77 (69–83) years; 3,031 (52%) were female, and 1,361 (23%) reported a history of dementia. Overall, 1,408 (24%) patients experienced incident delirium. In multivariable models, ED LOS was associated with development of delirium (odds ratio [OR] 1.02, 95% confidence interval [CI] 1.01–1.03, per hour), while non-clinical patient moves [OR 0.97, (95% CI 0.91–1.04) and ED hallway time [OR 0.99, 95% CI 0.98–1.01, per hour) was not associated with development of delirium.

**Conclusion:**

In this single-center study, ED length of stay was associated with incident delirium in older adults, while non-clinical patient moves and ED hallway time in the ED were not. Health systems should systemically limit time in the ED for admitted older adults.

## INTRODUCTION

Delirium, an acute, fluctuating condition with an alteration in level of consciousness associated with inattention and disorganized thinking, is the most common complication in hospitalized patients ≥65 years in age. It affects more than 2.6 million older adults each year with substantial annual costs for advanced healthcare systems, estimated to be between $38–152 billion in the United States (US).^2^–^4^ Delirium substantially impacts mortality, morbidity, and hospital length of stay (LOS).^5^,^6^ Delirium outcomes are worse in patients with dementia, and delirium may contribute to the development of dementia.^7^,^8^ Furthermore, preventing episodes of delirium may help to prevent dementia.^9^

Incident delirium is delirium that was not present on initial evaluation and develops during the hospital encounter. Known risk factors for incident delirium include sleep deprivation, lack of natural light, ambient noise, infection, immobility, urinary catheterization, malnutrition, history of cognitive impairment, pain, and acute medical conditions.^10^ Protective factors include early mobilization, maintenance of diurnal rhythms, and adequate hydration. The emergency department (ED) epitomizes a clinical space that is likely to precipitate delirium, especially if the exposure is prolonged or intense. As hospitals and EDs become more crowded, patients are spending more time in the ED. Early data from inpatient settings suggests that multiple bed moves are associated with increased delirium.^11^–^13^ However, it is unknown whether unnecessary non-clinical bed moves within the ED, in addition to the risks of longer ED LOS, are associated with development of incident delirium in older patients.

Our objective in this study was to evaluate the association between development of incident delirium during admission and (a) ED LOS, (b) ED hallway time, and (c) number of times a patient is moved from one treatment space to another for non-clinical reasons within the ED.

## METHODS

### Study Design and Setting

This retrospective cohort study was performed at a single, urban, academic ED with approximately 65,000 adult ED encounters annually. The study period extended from January 1, 2018–December 31, 2019. This study was approved by the local institutional review board.

### Study Population

We included consecutive patients ≥65 years presenting to the ED and admitted to the hospital on the internal medicine or family and community medicine services. Patients admitted to the ED observation unit prior to hospital admission were included in the study cohort. Patients were excluded if they were admitted to any other service, were admitted to an intensive care unit, were interfacility transfers from another health system, or were discharged directly from the ED or the ED observation unit. Also excluded were patients who underwent procedural sedation in the ED or as inpatients. We defined inclusion and exclusion criteria prior to data collection.

### Data Collection

All study data were directly extracted from the local electronic health record system (EHR) (Epic Systems Corporation, Verona, WI) by information technology (IT) data analysts. The IT data analysts were blinded to the study’s hypothesis and objectives. As part of the standard institutional data curation process, we validated key variables and a representative sample of complete records prior to final data extraction. Key variables validated included time intervals, number and type of bed moves, medication administration, restraint use, and delirium screen. The institutional data team had previously validated the Elixhauser Comorbidity Index (ECI) for data extraction. We did not manually review or abstract data for the final dataset. For this reason, using data abstraction forms, training and monitoring data abstractors, and measuring interobserver reliability as would be done for traditional chart review studies were not applicable.^14^

**Population Health Research Capsule**

What do we already know about this issue?

*Older adults are vulnerable to developing incident delirium during their emergency department (ED) stay*.

What was the research question?

*Is incident delirium associated with ED length of stay, time in the hallway, and number of bed movements?*

What was the major finding of the study?

*Length of stay in the ED was associated with development of incident delirium (OR 1.02, 95% CI 1.01–1.03, per hour)*.

How does this improve population health?

*Delirium is harmful, preventable, and costly to our healthcare system. Older adults should be given priority for bed assignment after admission*.

### Measurements

Key variables collected included patient demographics, ECI, history of dementia, time intervals in the ED, total number and type of patient movements in the ED, use of sedative medications, use of physical restraints, results of the Confusion Assessment Method–Intensive Care Unit (CAM-ICU) delirium screen, and encounter diagnoses. We calculated the ECI for each record according to the methodology described by van Walraven et al, and assigned the corresponding point value when a condition was present.^15^–^17^ The score ranges from −19 to 89, with a higher score indicating higher likelihood of in-hospital death.

We defined total ED LOS as the interval from ED arrival to physical departure from the ED; this included ED waiting time, ED treatment time, and any boarding time after the admission orders were placed in which the patient remained in the ED. Waiting time was defined as the interval from ED arrival to placement in a treatment bed or assignment of an attending physician to the patient, whichever occurred first. This included time spent in ED intake, triage, and waiting for a treatment bed. We defined ED treatment time as the interval from placement in an ED treatment bed to placement of inpatient bed request. Admit order time was defined as the interval from placement of an inpatient bed request to receipt of inpatient admission orders. Hallway time was defined as any time spent in a hallway bed. Throughout the study period, hallway beds were used only in the ED and not in the inpatient areas.

Patient moves were divided into clinical and non-clinical patient moves. We defined clinical patient moves as a patient changing physical locations between ED arrival and physical departure from the ED that directly advanced patient care. Examples include moving from ED triage to an ED treatment bed or from an ED treatment bed to ED radiology imaging. Non-clinical patient moves were defined as those that did not directly advance patient care. For example, moving from triage to any waiting area (waiting room or hallway waiting) or from one ED treatment bed to another was a non-clinical patient move.

Sedative medications included oral and parenteral benzodiazepines (lorazepam, midazolam, diazepam) and antipsychotic agents (haloperidol, olanzapine, risperidone, quetiapine) administered at any time during the patient’s ED or inpatient stay. Medications that were ordered but not administered were not included. Neither did we include antihistamines (diphenhydramine) or medications given for insomnia (melatonin, zolpidem).

The use of restraints was defined as an EHR order for any level of physical restraints during the ED or inpatient stay.

The CAM-ICU is the institutional delirium screen used in all levels of inpatient care. During inpatient care, the CAM-ICU was recorded by nursing staff twice daily. The CAM-ICU was variably recorded in the ED. Any positive CAM-ICU screen was considered to indicate the presence of delirium.

### Outcomes

Delirium was the primary outcome, defined as the composite outcome of a positive CAM-ICU screen at any time, administration of sedative medication, or use of patient restraints. We considered use of sedative medications and physical restraints to be a proxy for acute confusion, which equates to a positive CAM-ICU. Secondary outcomes included individual elements of the primary composite outcome: a positive CAM-ICU screen, administration of sedative medication, or use of physical restraints.

### Analysis

Analyses began with descriptive statistics. Logistic regression models with robust standard errors were fitted with the primary composite outcome as the dependent variable and the following independent variables: age; gender; English language preference; history of dementia; ECI, number of unnecessary non-clinical bed moves within the ED; total hallway time in the ED; ED LOS; and hospital LOS. We also fitted Poisson regression models with the same covariates with hospital LOS as the exposure, as the relationship between incident delirium and hospital LOS is bi-directional and complex. Patients missing outcome or predictor variables were excluded from analyses including the missing variables. We conducted all analyses using Stata 14 (StataCorp LP, College Station, TX).

## RESULTS

### Characteristics of Study Subjects

During the study period from January 1, 2018–December 31, 2019, 13,601 patients ≥65 years of age were admitted to our hospital. Sixty-six patients underwent procedural sedation during the admission and were excluded, resulting in a population of 13,535 patients. Of these, 5,886 patients were admitted from the ED to the internal medicine or family and community medicine service. This cohort included 3,031 (52%) women and 1,361 (23%) patients with a documented history of dementia. The study population included White non-Hispanic/Latinx (3,058; 52%), Black (802; 14%), Hispanic/Latinx (671; 11%), and Asian (601; 10%) patients (Table 1). No patients were excluded due to missing data.

### Main Results

Approximately one in four patients (1,408/5,886; 24%) experienced the primary composite outcome of a positive CAM-ICU screen, use of sedative medications, or use of physical restraints; 592 (10%) had a positive CAM-ICU screen; 1,086 (18%) received sedative medications; and 189 (3%) were physically restrained. After adjusting for demographic and clinical factors, ED LOS per hour was independently associated with both the primary composite outcome (odds ratio [OR] 1.02, 95% confidence interval [CI] 1.01–1.03) and the secondary outcomes of positive CAM-ICU screen (OR 1.02, 95% CI 1.00–1.02), administration of sedative medications (OR 1.02, 1.01–1.02), and use of physical restraints (OR 1.02, 95% CI 1.01–1.02) (Table 2). Male patients were more likely to receive physical restraints (OR 1.40, 95% CI 1.03–1.89) and less likely to receive sedative medication (OR 0.85, 95% CI 0.75–0.98) compared to female patients. Higher ECI score was associated with all outcomes (Table 2). The number of ED non-clinical patient moves were not associated with the primary composite outcome (OR 0.97, 95% CI 0.91–1.04) or secondary outcomes (Table 2).

## DISCUSSION

Delirium is a common, expensive to treat, and partially preventable condition in older adults that is under-recognized and may have devastating sequelae.^4^,^18^ The ED environment may promote the development of incident delirium.^19^–^21^ Associations between ED LOS and incident delirium have been recently described.^1^,^22^ Our study also found ED LOS associated with the development of incident delirium.

Multiple room transfers have been associated with incident delirium and with falls in the ED^1^ and inpatient setting.^11^–^13^ The current study of older adults did not find a significant association between the development of incident delirium and non-clinical patient moves within the ED. However, only 11% of our cohort underwent three or more non-clinical patient moves. It is possible that the low number of non-clinical patient moves in our cohort mitigated the development of incident delirium. Our study of older adults did not find an association between time spent in the ED hallway and incident delirium in contrast to a prior study.^18^

Multiple screening tools for delirium exist^23^; in this study we used the CAM-ICU delirium screening tool built into the institution’s EHR. The feasibility of screening for delirium in the ED and in the inpatient setting remains complex. Clinician gestalt without a formal screening tool is associated with poor sensitivity and specificity.^21^,^23^ The CAM-ICU is brief, easy to administer, and has been shown to have excellent specificity in older adult ED patients (although specificity decreases in patients who have dementia).^24^ Both sedative medications and restraints are more frequently used when behaviors associated with hyperactive delirium are present such as agitation or attempts to get out of bed. It is likely that patients with hypoactive or mixed delirium are undercounted using this primary composite model, as it is easier to clinically recognize hyperactive delirium than hypoactive delirium. Prior research in the ED suggests that hyperactive delirium accounts for less than 10% of ED delirium, while hypoactive or mixed delirium is more common, less likely to be recognized, and accounts for substantial mortality.^18^,^25^

Approximately 10% of patients in this study screened CAM-ICU positive. English-speaking patients were more likely to have a positive CAM-ICU screen. The CAM-ICU requires excellent English comprehension; patients who do not have conversational English may be disadvantaged, even with liberal use of interpreters.

Nearly 20% of older inpatients admitted through the ED received a sedative medication at some point during their hospitalization. With known exceptions, such as alcohol or benzodiazepine withdrawal, sedative medications do not treat the underlying processes precipitating delirium. Of note, we found that female patients were significantly more likely to have sedative medications administered. In contrast, male patients were more likely to be physically restrained. To our knowledge, there are no prior studies that have reported gender differences in the management of delirium in older adults.

Physical restraints were used in 3% of older adults in this study, most commonly in patients with history of dementia and in male patients. This proportion is substantially lower than recent reports showing restraint use in hospitalized non-critical care patients to be between 8.5–11.8%.^26^,^27^ Restraints have not been shown to reduce falls and may increase the risk of developing delirium in hospitalized patients,^26^,^28^ highlighting the importance of minimizing restraint use in older adults.

## LIMITATIONS

This retrospective cohort study was performed at a single, urban, academic hospital, and our experiences may differ from those in other institutions. While this study has the limitations inherent in a retrospective cohort study, it is strengthened by adherence to applicable methodologic recommendations.^14^ During the study period, delirium screening was not consistently performed in the ED, limiting our ability to identify delirium that was present on arrival. This study did not adjust for receipt of opioids or adequacy of pain control measures. Other risk factors for developing delirium that were not routinely documented in the EHR included living in a residential care facility, sensory impairments such as hearing or vision loss, and outpatient polypharmacy.

## CONCLUSION

This study builds on recent work that suggests prolonged ED length of stay is harmful for older patients who require admission. Longer ED LOS (per additional hour) was significantly associated with the development of delirium in older patients admitted to the hospital. Patients and health systems will benefit if admitted older patients, especially those with a history of dementia and multiple comorbidities, are promptly assigned and moved to a hospital inpatient bed, minimizing their length of stay in the ED.

## Figures and Tables

**Table 1:** Demographic and clinic characteristics (N=5,886).

Characteristic	N (%)
Age*	77 (69, 83)
Female gender	3,031 (51%)
Race/ethnicity	
White	3,058 (52%)
Black	802 (14%)
Hispanic or Latinx	671 (11%)
Asian	601 (10%)
Multiracial/other	709 (12%)
Not available	45 (1%)
English-language preference	4,799 (81%)
History of dementia	1,361 (23%)
Elixhauser Comorbidity Index*	18 (10, 26)
ED length of stay (hours)*	16 (8.0, 21)
ED wait time (hours)*	1.2 (0.1, 1.6)
ED treatment time (hours)*	4.1 (2.2, 4.8)
ED hallway time (hours)*	1.5 (0.6, 4.7)
Total ED non-clinical patient moves	
0	1,172 (20%)
1	2,413 (41%)
2	1,625 (28%)
3 or more	676 (11%)
Positive CAM-ICU screen	592 (10%)
Use of physical restraints	189 (3%)
Use of sedative medication	1,086 (18%)

* Data presented as median (25th, 75th percentile) *ED*, Emergency department; *CAM-ICU*, Confusion Assessment Method.

**Table 2:** Multivariable logistic regression results.

Variable	Primary outcome	Secondary outcomes		

	Incident delirium N=1,408	Positive CAM-ICU screen, N=592	Sedative medication N=1,086	Physical restraint use N=189
Age (per year)	0.98 (0.98–0.99)	1.02 (1.02–1.09)	0.97 (0.96–0.98)	1.00 (0.98–1.02)
Male gender	0.86 (0.75–0.97)	1.01 (0.84–1.21)	0.85 (0.75–0.98)	1.40 (1.03–1.89)
English language	1.72 (1.45–2.05)	1.33 (1.05–1.69)	1.95 (1.59–2.38)	0.81 (0.56–1.17)
History of dementia	2.86 (2.48–3.30)	3.00 (2.49–3.63)	2.34 (1.99–2.73)	4.64 (3.35–6.42)
Elixhauser Comorbidity Index (per 10 points)	1.02 (1.01–1.02)	1.03 (1.03–1.04)	1.01 (1.00–1.01)	1.02 (1.01–1.03)
Total ED non-clinical bed moves	0.97 (0.91–1.04)	1.00 (0.91–1.10)	0.99 (0.92–1.06)	1.15 (0.98–1.34)
ED hallway time (per hour)	0.99 (0.98–1.01)	1.00 (0.98–1.02)	0.99 (0.98–1.01)	1.00 (0.97–1.03)
ED LOS (per hour)	1.02 (1.01–1.03)	1.02 (1.00–1.02)	1.02 (1.01–1.02)	1.02 (1.01–1.02)

*CAM-ICU*, Confusion Assessment Method; *ED*, emergency department; *LOS*, length of stay.

Data are presented as odds ratios with 95% confidence intervals.
